# Structure-Function Relationship of TRPM2: Recent Advances, Contradictions, and Open Questions

**DOI:** 10.3390/ijms21186481

**Published:** 2020-09-04

**Authors:** Frank J.P. Kühn

**Affiliations:** Institute of Physiology, University Hospital RWTH Aachen, 52074 Aachen, Germany; fkuehn@ukaachen.de

**Keywords:** transient receptor potential, cryogenic electron microscopy, adenosine diphospho ribose, NUDT9, ion channel orthologous

## Abstract

When in a particular scientific field, major progress is rapidly reached after a long period of relative stand-still, this is often achieved by the development or exploitation of new techniques and methods. A striking example is the new insights brought into the understanding of the gating mechanism of the transient receptor potential melastatin type 2 cation channel (TRPM2) by cryogenic electron microscopy structure analysis. When conventional methods are complemented by new ones, it is quite natural that established researchers are not fully familiar with the possibilities and limitations of the new method. On the other hand, newcomers may need some assistance in perceiving the previous knowledge in detail; they may not realize that some of their interpretations are at odds with previous results and need refinement. This may in turn trigger further studies with new and promising perspectives, combining the promises of several methodological approaches. With this review, I aim to give a comprehensive overview on functional data of several orthologous of TRPM2 that are nicely explained by structural studies. Moreover, I wish to point out some functional contradictions raised by the structural data. Finally, some open questions and some lines of possible future experimental approaches shall be discussed.

## 1. Overview

Originally discovered around the turn of the millennium under its former names TRPC7 or LTRPC2, the human TRPM2 (transient receptor potential melastatin type 2 cation channel; hTRPM2) channel quickly caught broad scientific interest [1,2,3]. Besides TRPM7/TRPM6 [4,5] it represents the only TRP channel containing a C-terminal enzyme domain [2]. This domain with strong homology to the human nucleotide diphosphate linked moiety X type (Nudix) hydrolase motif 9 (NUDT9) essentially determines the physiological role of hTRPM2. ADP-ribose (ADPR), the natural substrate of the NUDT9 enzyme [6] represents one of several metabolites of β-NAD+, a major component of cellular bioenergetics and signaling pathways. After ADPR was identified as the principal agonist of hTRPM2 [2,3] it was soon recognized that this ion channel represents a key player in the process of oxidative stress-mediated apoptosis [2,7,8]. Since then, numerous studies have been produced showing the involvement of hTRPM2 in diverse pathophysiological processes including cancer, CNS-pathologies, NLRP3-inflammasome activation, warmth sensation and diabetes mellitus e.g., reviewed in [9,10,11,12,13]. Based on these findings, it is not surprising that the exploration of the gating mechanism of hTRPM2 has been performed with great effort being constantly spurred on by the challenging complexity of this process. In this review, I want to summarize and condense the currently available data to facilitate the development of further experimental strategies which are necessary to elucidate channel gating of TRPM2.

## 2. The Long Standing Paradigm of ADPR-Directed Channel Gating of Human TRPM2

A reasonable first hypothesis for the gating mechanism of TRPM2 was quickly derived from the presence of a C-terminal domain with strong homology to a cytosolic ADPR hydrolase (ADPRase) together with the experimental finding that the channel can be activated by ADPR [2]. As a result, ADPR binds to the NUDT9 homology domain (NUDT9H) and induces pore opening via conformational rearrangements of the channel protein [2]. The crucial question however, was whether or not this process required the hydrolysis of ADPR (comparable to the well-established hydrolysis of ATP observed in many other proteins). Because of specific sequence differences between NUDT9H and the native NUDT9 enzyme, for hTRPM2 an impaired ADPRase activity was assumed [2]. This conclusion was supported by experimental data where the ADPRase activities of NUDT9 enzyme and NUDT9H domain of hTRPM2 were compared in vitro [2,14,15]. A series of experiments performed by Kühn et al. [16] and Csanády and co-workers [17,18] provided further evidence to support this hypothesis. Most importantly, it was shown that the non-hydrolyzable ADPR analog α-β-methylene-ADPR (AMPCPR) also activates the channel [17]. In addition, using in vitro ADPRase assays it was confirmed that the purified NUDT9H fragment of hTRPM2 lacks significant catalytic activity [18]. Nonetheless, the degenerated catalytic center of NUDT9H must have critical importance for hTRPM2 since it was demonstrated that restoration of the canonical sequence prevents channel gating [8,19].

Much effort has been invested to clear up the physiological pathway for the ADPR-dependent activation of hTRPM2 in vivo. It has been shown that oxidative stress induces intracellular pathways leading to poly-ADP-ribosylation of DNA and various nuclear proteins. This process is catalyzed by the enzyme Poly-ADPR Polymerase type 1 (PARP-1). Subsequently, Poly-ADPR-Glycohydrolase (PARG) degrades the poly-ADPR chains to monomeric ADPR e.g., reviewed in [20]. The resulting intracellular accumulation of ADPR induces the activation of hTRPM2 [8]. These findings explain earlier observations where the extracellular application of hydrogen peroxide onto hTRPM2-expressing cells also induces the characteristic ADPR-dependent currents [2,7,21]. A direct functional effect of hydrogen peroxide on TRPM2 channels could be excluded with inside-out patch-clamp studies where even the prolonged exposure to high concentrations of hydrogen peroxide failed to activate or inhibit hTRPM2 [22].

Experimental studies in macrophages show clear evidence for a functional relationship between the hydrogen peroxide-dependent activation of hTRPM2 and body temperature [22]. Evidently, in these cells an increased level of hydrogen peroxide reduces the threshold for temperature-dependent channel activation. A single methionine residue (Met-215), which is located within the N-terminal domain of hTRPM2, plays a crucial role in this process [23]. Interestingly, the latest structure function studies indicate that in TRPM2 channels the adjacent region of the critical methionine residue is of central importance for ADPR-dependent channel function [24,25,26,27]. However, a direct effect of hydrogen peroxide on this methionine residue has not yet been demonstrated. In the context of co-stimulation of hTRPM2 by oxidative stress and temperature, cyclic ADPR (cADPR) has been repeatedly suggested as a potential agonist or at least co-regulator of ADPR-dependent channel gating. However, the experimental data collected so far are inconsistent [25,28,29,30,31,32,33,34]. One major problem with commercially available cADPR is that it is often contaminated with ADPR [22,33]. Therefore, a putative stimulation by cADPR actually could be an ADPR-dependent activation. Nevertheless, a recent study [34] on hTRPM2 strongly suggests an agonistic function of cADPR taking into account all of the possible experimental pitfalls (i.e., contamination by ADPR, intracellular degradation of cADPR, indirect activation mechanisms of cADPR via intracellular signaling pathways). It remains to be clarified whether cADPR also acts as an agonist for further TRPM2 orthologues.

In contrast, there is general agreement about the important role of calcium cations (Ca^2+^) as an essential cofactor of ADPR-dependent activation of hTRPM2 [31,35,36,37,38]. The stimulatory effect of Ca^2+^ on ADPR-evoked currents of TRPM2 is due to a positive feedback mechanism [31,35], where Ca^2+^ enters the pore of already activated channels to ensure the saturation of intracellular binding sites [38,39]. A common feature of the TRPM subfamily is the regulation by phosphatidylinositol 4.5 bisphosphate (PIP_2_) e.g., reviewed in [40,41]. This process has been intensively studied especially for TRPM8 which represents the closest relative of TRPM2 within the superfamily of TRP cation channels [42,43,44]. In a study by Tóth and Csanády [45] it was found that PIP_2_ has a positive effect on the ADPR/Ca^2+^-dependent activation of hTRPM2. This was evident after removal of naturally occurring PIP_2_ and subsequent replenishment via artificial PIP_2_. These experiments were carried out with inside-out patches of *Xenopus* oocytes and unusually high concentrations of Ca^2+^ (up to 125 µM) were used. It was suggested that both Ca^2+^ and PIP_2_ stabilize the open state of the channel or alternatively that PIP_2_ is directly involved in Ca^2+^-binding [39].

Significant progress was achieved in characterizing the pharmacological profile of hTRPM2 [46,47,48]. ADPR-2-phosphate (ADPR-2P) and 2′ deoxy-ADPR (2d-ADPR) were identified as further agonists of hTRPM2, perhaps with physiological potential [49,50]. On the other hand, a series of effective antagonists of ADPR-dependent channel gating of hTRPM2 were developed. These antagonists are exclusively synthetic ADPR derivatives [46,51].

With regard to the exploration of the detailed functional effect of the principal agonist ADPR on hTRPM2, for a long time the main focus was solely limited to the NUDT9H domain [2,8,18,19,52]. This seemed obvious, since this domain is homologous to an enzyme that binds and hydrolyzes ADPR [6]. However, in the absence of any detailed structural information of the entire channel protein, the structural and functional impact of ADPR-binding to NUDT9H could not be determined.

## 3. Paradigms Shifted—Discovery of a Novel ADPR-Dependent Gating Mechanism

The structure function study of proteins by the comparative analysis of species-specific variations represents a classical experimental approach that has been also successfully applied in the field of TRP ion channels e.g., [53,54]. For TRPM2, however, for a long time the focus was exclusively on mammalian variants, especially on the human orthologue. A first paradigm shift was largely inspired by a study of Mederos ySchnitzler et al. [55] in which an exciting hypothesis was made. They found that in basal metazoans (e.g., *Nematostella vectensis*) and unicellular protists (e.g., *Monosiga brevicollis*) there is only one representative of the TRPM subfamily, which could be easily identified as TRPM2-like. This TRPM2-variant was proposed as the potential evolutionary ancestor of all members of the TRPM subfamily currently existing in mammals, a hypothesis which certainly swept away all the assumptions that had prevailed up to that point.

In 2014 a project was started to functionally characterize such a putative TRPM2 ancestor by heterologous expression in HEK-293 cells. A species variant of the sea anemone *Nematostella vectensis* (nvTRPM2) was selected, which displays a total sequence identity of 31% to the corresponding open reading frame of hTRPM2. *Nematostella vectensis* represents a well-established model organism with fully characterized genome sequence [56]. An even more important feature of nvTRPM2 is given by its remarkable protein sequence of the NUDT9H domain. This sequence shows much more identity with the human NUDT9 enzyme, if compared with the NUDT9H-domain of hTRPM2 [57]. Most importantly, the putative active site of the human NUDT9 enzyme containing the characteristic Nudix box signature GX5EX7REUXEEXGU [58] is almost completely preserved in nvTRPM2 but significantly modified in hTRPM2 [8,57]. This feature strongly suggests a largely intact catalytic function for the NUDT9H domain of nvTRPM2, but, according to the prevailing doctrine at that time, this should be actually incompatible with proper channel function e.g., [2,8,19]. However, the electrophysiological analyses revealed that heterologous expression of nvTRPM2 in HEK-293 cells generates functional cation channels, which can be fully activated by application of ADPR via the patch pipette [57]. The presence of the co-agonist Ca^2+^ at least on one side of the cell membrane was found to be essential for nvTRPM2 as already described for hTRPM2 e.g., [37]. However, the current potentiation of nvTRPM2 induced by Ca^2+^ was significantly less pronounced, if compared with hTRPM2 [57].

A striking difference between hTRPM2 and nvTRPM2 becomes apparent by the different current kinetics of whole-cell patch-clamp recordings during stimulation with moderate concentrations of ADPR. While the current onset of hTRPM2 develops slowly, reaching maximum amplitudes after 1–2 min, nvTRPM2 shows maximum currents within a few seconds after establishing whole-cell configuration. The slow current onset of hTRPM2 in whole-cell configuration is particularly prominent during stimulation with threshold concentrations of ADPR (e.g., during stimulation with hydrogen peroxide, when intracellular accumulation of ADPR represents the limiting factor) or when the intracellular concentration of Ca^2+^ is strongly reduced by Ca^2+^-chelators. On the other hand, maximum currents of nvTRPM2 return to baseline levels almost as quickly as they are generated, while hTRPM2 exhibits a gradual current decline that can last for several minutes [57]. Remarkably, in inside-out patch-clamp measurements the respective current inactivation kinetics of the two TRPM2 species variants were found to be exactly the opposite. In this case, nvTRPM2 remains open while hTRPM2 quickly inactivates [39]. There is currently no satisfactory explanation for this striking discrepancy of data obtained under different experimental conditions.

One of the most surprising findings was that currents of nvTRPM2 could not be stimulated with hydrogen peroxide which initially was difficult to reconcile with the observation that nvTRPM2 is far more responsive to ADPR than hTRPM2 [57].

In light of the observed differences between the two distantly related species variants of TRPM2 with regard to the catalytically active site of NUDT9H on one side and the sensitivity to hydrogen peroxide on the other side, it was tempting to functionally characterize chimeras of hTRPM2 and nvTRPM2 containing swapped NUDT9H domains. As a result it was found that neither ADPR nor hydrogen peroxide can stimulate the hTRPM2 variant containing the NUDT9H domain of nvTRPM2. In sharp contrast, the nvTRPM2 channel variant with the NUDT9H domain of hTRPM2 was shown to be fully sensitive to ADPR and additionally sensitive to hydrogen peroxide [57]. Similar results were obtained with chimeras of both channel orthologues where the original NUDT9H-domain was replaced by the corresponding sequence of the human NUDT9 enzyme [57]. These data strongly suggest a highly sophisticated functional interaction between channel domain and endogenous NUDT9H in wild-type hTRPM2, whereas channel function of nvTRPM2 seemed to be far more flexible in this regard. In further experiments it was possible to link this functional effect to specific sequence regions of NUDT9H or even to individual point mutations [16,57]. After the discovery that the identity of the NUDT9H domain does not significantly affect the ADPR-dependent gating of nvTRPM2, the next logical step was to test whether this domain is necessary for the channel function of nvTRPM2 at all. Therefore, the corresponding variants of hTRPM2 and nvTRPM2 with deleted (Δ) NUDT9H domain were generated and both surface expression and channel function were analyzed during heterologous expression in HEK-293 cells [16]. As a result, a loss of function phenotype for properly surface-expressed hTRPM2-ΔNUD as well as a fully functional channel variant nvTRPM2-ΔNUD with largely unaffected sensitivity to ADPR and additional sensitivity to hydrogen peroxide were identified [16]. The latter result revealed that ADPR-dependent activation of TRPM2 in principle can be accomplished independently of the NUDT9H domain and thus challenged the previously adopted standard hypothesis [59]. In addition to this main finding, further interesting results were obtained from these initial studies on nvTRPM2 as briefly summarized in the following:

A triple hemagglutinin tag attached to the C-terminus selectively disrupts the structure-function relationship of the NUDT9H domain [16]. The data obtained from co-expression experiments of NUDT9H and nvTRPM2-ΔNUD strongly suggest that the NUDT9H domain of nvTRPM2 must have sizeable ADPRase activity [16]. A point mutation within the N-terminal region of NUDT9H is of essential functional importance both for hTRPM2 and nvTRPM2 [16]. 2-Aminoethoxydiphenylborate (2-APB), an established inhibitor of hTRPM2 [60], proves to be a Ca^2+^-dependent activator of nvTRPM2, which also prevents channel inactivation [61]. All these findings provided important indications for a novel type of gating mechanism present in nvTRPM2 but in the absence of reliable structural data the solid basis for establishing a novel model of ADPR-dependent channel gating was missing.

## 4. The Cryo-EM-Era—Different Species, Similar Structures, but Different Mechanisms?

With the publication of the cryogenic electron microscopy (cryo-EM)-structure of TRPV1 [62] a new era of structure-function analysis had been started in the field of TRP channel research [63]. Afterwards, in rapid succession, more and more TRP channel structures were characterized until the first member of the more complex TRPM subfamily was deciphered [64]. Interestingly, within the TRPM2 field which most of the time had focused mainly on the human variant, the first cryo EM structures were published on nvTRPM2 [39] and zebrafish TRPM2 (drTRPM2) [24]. The cryo EM structure of nvTRPM2 was limited to the channel domain, because the NUDT9H domain could not be successfully mapped [39]. Unfortunately, no N-terminal ADPR binding pocket was identified in this cryo-EM structure, and therefore an in-depth characterization of the novel ADPR-dependent gating mechanism of nvTRPM2 could not be performed. However, the finding that the NUDT9H-domain is very flexibly linked to the channel structure [39] fits very well to previous data, which demonstrated functional independence of the NUDT9H domain [16,59].

Then, however, a novel ADPR binding site was identified within the N-terminal part of drTRPM2, but the functional characterization also showed that the endogenous NUDT9H domain is still indispensable for channel activity [24]. Notably, the N-terminal ADPR interaction site represents the only one that was detected in the published structure of drTRPM2 [24]. The proposed amino acid residues involved in ADPR binding are highly conserved in all species variants of TRPM2, although the parent domain is characteristic for all members of the TRPM subfamily [65,66,67]. Interestingly, the data of Huang et al. [24] strongly suggest that mutations with the most negative effect on ADPR-dependent channel activity (deletion of NUDT9H; double mutation R278A+R334A) also show the lowest expression levels. Therefore, it could not be excluded that the evaluated mutants might have additional non-specific effects on channel structure.

The third cryo-EM analysis of a TRPM2 channel, albeit with limited resolution, finally delivered extensive structural data for human TRPM2 [68]. In this structure, instead of an N-terminal binding pocket, the classic ADPR binding site within the NUDT9H domain was identified. Moreover, data were presented where the corresponding double arginine to alanine (RR/AA) mutation of the postulated N-terminal ADPR binding pocket did not induce significant functional effects in hTRPM2. Instead, it was demonstrated that this double mutation substantially reduces the surface expression of hTRPM2 [68]. Furthermore, in the same study it was shown that the binding affinity of ADPR to the respective NUDT9H domain is decreased in drTRPM2 by a factor of about 25, if compared with hTRPM2. This finding strongly suggests that binding of ADPR to the NUDT9H domain does not play a major role for channel gating of drTRPM2 [68]. Thus, at the end of 2018, the available structure-function data provided a very confusing picture of the gating mechanism of TRPM2. This shortcoming should improve significantly in the course of the following year.

## 5. TRPM2—A Channel with Two Distinct ADPR Binding Pockets

In 2019, two additional cryo EM structures were published. The principal findings of the currently available cryo EM studies are summarized in Table 1. For drTRPM2 the structural rearrangements during ligand binding were characterized in more detail and the presence of twofold-symmetric intermediate gating states was proposed [69]. Furthermore, a new high-resolution cryo EM structure of hTRPM2 was presented, which now contains two separate ADPR binding pockets [25], the classic NUDT9H binding site and the novel N-terminal binding pocket as already identified in drTRPM2.

In this latest structure of hTRPM2, for the first time ADPR was detected in both binding pockets and additionally 8-bromo (Br)-cADPR was found to bind selectively to the N-terminal binding pocket [25]. Such a pharmacological differentiation of the two different ADPR binding pockets was previously described for nvTRPM2 [70]. In this study it was demonstrated that both 8-(thiophen-3-yl)-ADPR (8-TP-ADPR) and 8-(3-acetyl-phenyl)-ADPR (8-(3AcPhe)-ADPR), which represent strong antagonists of hTRPM2 [46], readily activate nvTRPM2-ΔNUD, and therefore must bind to the N-terminal binding pocket. Moreover, IDP-ribose (IDPR), an ADPR-analogue that only contains a small modification of the adenine ring at C−6, neither activates nor inhibits nvTRPM2-ΔNUD, even when applied in high concentrations. In contrast, full-length nvTRPM2 was already stimulated with moderate concentrations of IDPR [59]. Since IDPR was shown previously to serve as a substrate for the native NUDT9 enzyme [6] and it was also demonstrated that hTRPM2 can be stimulated with high concentrations of IDPR [70], the most plausible assumption is as follows:

IDPR does not effectively interact with the N-terminal binding pocket but it can stimulate channel activation via the binding pocket of NUDT9H, both in hTRPM2 and in nvTRPM2. In case of nvTRPM2, IDPR-dependent activation takes place indirectly via competitive inhibition of the NUDT9H-associated ADPRase which leads to intracellular accumulation of ADPR. This mechanism is quite similar to the activation of NUDT9H-defective variants of nvTRPM2 during stimulation with hydrogen peroxide and in both cases is reflected by characteristically delayed current kinetics [70].

In view of the currently available data, the observed stimulation of hTRPM2 with IDPR is more difficult to explain. If, according to the results of Huang et al. [25], both binding pockets must be occupied with ADPR to stimulate hTRPM2, then IDPR should not activate the channel because it exclusively operates via the NUDT9H domain. A possible way out of this dilemma would be to assume that the substrate specificities of the corresponding N-terminal binding sites are different in hTRPM2 and nvTRPM2. However, at least for nvTRPM2 and drTRPM2 this is definitely not the case as demonstrated with 8-TP-ADPR and 8-(3AcPhe)-ADPR [26,70]. For the same reason IDPR cannot directly activate drTRPM2 via the N-terminal binding pocket [26]. However, in contrast to nvTRPM2, the NUDT9H domain of drTRPM2 does not even bind ADPR effectively and most probably is catalytically inactive [68]. Consequently, indirect channel activation mediated by IDPR, as demonstrated for nvTRPM2 [70], was not detected in drTRPM2 [26].

For several ADPR analogues, there are also similarities between hTRPM2 and nvTRPM2 with regard to the pharmacological profile. For example, ADPR-2-phosphate, 2-Fluor-ADPR [16,70] as well as AMPCPR and 2-deoxy-ADPR [17,27], activate both channels to about the same extent, while ADP-glucose, β-methyl-ADP and cyclopentyl-ADP have no significant effects [47,50,70]. Notably, the finding of Tóth et al. [27] that 8-Br-ADPR, representing an established inhibitor of hTRPM2 [46], selectively activates nvTRPM2 nicely confirms previous results [46,70], where the opposite effects of 8-substituted ADPR analogs on these two channel orthologous were originally described. The nvTRPM2 channel is particularly well suited for determining the pharmacological profiles of channel domains and NUDT9H, since these two domains are functionally independent [16]. For this purpose, two experimental approaches were used: Kühn et al. [70] compared agonist sensitivities of wild-type hTRPM2, full-length nvTRPM2 and nvTRPM2-ΔNUD, while Tóth et al. [27] examined channel activation of wild-type nvTRPM2 as well as the ADPRase activity of the separately purified NUDT9H domain. A summary of the currently available data on the substrate specificities of the two different ADPR binding pockets of TRPM2 is given in Table 2.

Altogether, the double ADPR binding hypothesis for hTRPM2 raises a number of further questions: Since 8-Br-cADPR neither inhibits ADPR-dependent activation nor binds to the NUDT9H domain [25], ADPR should bind much more effectively to the N-terminal binding pocket than 8-Br-cADPR. However, the cryo EM data of Huang et al. [25] strongly suggest that the assumed U-shaped conformation of 8-Br-cADPR does fit far better into the small N-terminal binding pocket than ADPR. Furthermore, in the same study it was postulated that 8-Br-cADPR selectively inhibits cADPR-dependent channel gating—but where does cADPR actually bind? In the cryo EM structure of Huang et al. [25] the binding of cADPR remained undefined, whereas previous studies on hTRPM2 postulated that cADPR binds to NUDT9H [34].

The two nvTRPM2 agonists 8-TP-ADPR and 8-(3AcPhe)-ADPR were clearly shown to activate nvTRPM2 independently of the NUDT9H domain [70]. Their respective substituents at position 8 are significantly larger than the bromine atom in 8-Br-cADPR but obviously they still fit nicely into the narrow N-terminal binding pocket. For these two agonists of nvTRPM2 which also represent effective antagonists of hTRPM2 [46], the molecular conformation has not been described yet. However, the U-shaped conformation is rather unlikely. According to the interpretation of Huang et al. [25] it is also conceivable that the antagonistic effects of 8-TP-ADPR and 8-(3AcPhe)-ADPR on hTRPM2 were not mediated by NUDT9H, as previously assumed [46], but rather via the N-terminal binding pocket. However, once again this assumption would imply that the same ligands have opposite effects at the highly conserved N-terminal ADPR binding pockets of hTRPM2 and nvTRPM2. In light of the data obtained for nvTRPM2 and drTRPM2 [26] this hypothesis seems rather unlikely. Thus, 8-TP-ADPR and 8-(3AcPhe)-ADPR are likely to inhibit hTRPM2 via the NUDT9H domain and stimulate nvTRPM2 and drTRPM2 via the N-terminal binding pocket. Since for nvTRPM2 and drTRPM2 ligand binding to the NUDT9H domain is largely irrelevant for channel activation, the agonistic effect via the N-terminal binding pocket dominates. This scenario seems to be the case even with the chimera drTRPM2-hNUD [26]. In this variant, the N-terminal binding site of drTRPM2 was combined with the NUDT9H domain of hTRPM2.

As a result, the chimera should have two intact ADPR binding pockets, making it more similar to hTRPM2 than to drTRPM2. The functional analysis of drTRPM2-hNUD shows an ADPR-dependent activation which is very similar to that of wild-type drTRPM2. Moreover, when the chimera was stimulated with 8-(3AP)-ADPR, full channel activation was induced [26], while complete inhibition was observed in hTRPM2 [46]. Thus, in contrast to hTRPM2, the N-terminal binding pocket apparently dominates in drTRPM2-hNUD. What might be the reason for this difference between hTRPM2 and drTRPM2-hNUD?

In hTRPM2 the 8-substituted ADPR analogues putatively bind to NUDT9H as well as to the N-terminal binding pocket which, according to Huang et al. [25], both contribute to channel activation. Hence, the experimental finding that 8-TP-ADPR and 8-(3AcPhe)-ADPR inhibit hTRPM2 [46] could indicate a dominant role of NUDT9H for channel gating. The pharmacological profiles of both ADPR binding pockets are probably not as different as it seems because the species-specific functional effect of the NUDT9H domain might represent the crucial factor. Currently only IDPR was shown to bind exclusively to the NUDT9H domain [70], which is quite remarkable given the minimal structural difference between IDPR and ADPR.

With regard to the proposed universal N-terminal binding pocket there are also some structural inconsistencies which are difficult to explain in light of the observed pharmacological profiles of the different species variants. The amino acid sequence which forms the proposed N-terminal binding pocket of drTRPM2 varies in only one position if compared to hTRPM2 and nvTRPM2, respectively. Each of these variations represents a quite conservative mutation (e.g., threonine to serine) and yet the pharmacological profile between hTRPM2 on the one hand and drTRPM2 and nvTRPM2 on the other hand is shown to be fundamentally different. In contrast, the corresponding sequence of the species variant of the invertebrate *Salpingoeca rosetta* (srTRPM2) differs from the consensus sequence in two critical positions (tyrosine to phenylalanine and arginine to leucine). Nevertheless, ADPR-dependent gating of this species variant is comparable to that of nvTRPM2 and is also independent of the NUDT9H domain [71]. Moreover, the consensus sequence of the putative N-terminal ADPR binding pocket is also conserved in the human cold and menthol receptor TRPM8, although this channel was found to be insensitive to ADPR, even with C-terminally attached NUDT9H domain [72].

The consensus sequence of the N-terminal ADPR binding pocket present in hTRPM2, drTRPM2 and nvTRPM2 was extensively analyzed with site-directed mutagenesis. Whereas the loss-of-function effect of the RR/AA double mutation within the N-terminal binding pocket is consistently observed in drTRPM2, nvTRPM2 and hTRPM2 [24,25,26], divergent results were obtained with alanine substitution scanning analyses of single amino acid residues. The functional effects examined with inside-out patch clamp recordings of single alanine substitution mutants were found to be relatively moderate in drTRPM2 (e.g., Y271A, R334A) [24] while the corresponding mutations (Y264A, R329A) in nvTRPM2 showed much stronger impact when tested under similar experimental conditions [27]. Correspondingly, in hTRPM2 the impact of the mutations Y295A and R302A were found to be much stronger than those induced by Y271A and R278A in drTRPM2 [24,25]. In any case, for the NUDT9H domain of hTRPM2, as a genuine ADPR binding pocket, the loss-of-function phenotype by virtue of alanine substitutions of critical amino acid residues (H1346A, T1347A, Y1349A, L1379A, D1431A, R1433A, H1488A) was much more frequently observed [52], if compared with the proposed N-terminal ADPR-binding pocket of TRPM2 [24,25,27].

Meanwhile, the loss-of-function phenotype of the critical RR/AA double mutation originally characterized in drTRPM2 [24] was also confirmed for hTRPM2 and nvTRPM2 [25,26]. Moreover, a misinterpretation of the functional data by different levels of surface expression of the mutants could be excluded [26].

Most importantly, another result of the same study [26] does not quite fit the hypothesis of a uniform N-terminal ADPR binding pocket. As previously shown for nvTRPM2 [61], drTRPM2 can be stimulated with 2-APB and this stimulation was suppressed by the RR/AA double mutation [70]. The interaction between TRPM2 and 2-APB has not yet been characterized in any of the published cryo-EM structures, and therefore there is no structural information that could support a molecular interaction of both activation mechanisms. However, this result could also reflect a rather unspecific effect of the double mutation which is localized in the melastatin homology region (MHR), a highly conserved parent domain of all members of the TRPM subfamily [65,66,67].

## 6. NUDT9H—Species-Specific Functional Characteristics and Evolutionary Aspects

Regardless of the rapid progress recently made to pinpoint the structure-function relationship of TRPM2, the central question remains unanswered: In exactly which way does the NUDT9H domain control ADPR-directed channel activation? Answering this question seems to have become even more complicated after characterizing different species variants of TRPM2. With regard to nvTRPM2, the interaction between channel domain and NUDT9H is relatively simple—there is no interaction at all [16,27]. Nevertheless, the NUDT9H domain of nvTRPM2 may have an indirect regulatory role in vivo due to its fully developed ADPRase activity. Currently, one can only speculate as to what this regulation actually looks like in vivo, but some experimental findings may already point into the right direction. This could be for example a competitive inhibition of the ADPRase activity as shown with IDPR [70] or some kind of feedback regulation by TRPM2-mediated influx of Mg^2+^ [71]. Both Mn^2+^ and Mg^2+^ represent important cofactors for the enzymatic activity of the human NUDT9 enzyme. In the presence of hydrogen peroxide, this functional role is shifted from Mg^2+^ to Mn^2+^ [73]. Possibly, such a mechanism may be also physiologically important for nvTRPM2 in vivo [16].

A completely different picture emerges for the two vertebrate TRPM2 channels hTRPM2 and drTRPM2. In both cases, channel activity is completely lost after removal of the NUDT9H domain [16,24]. The comparative sequence analysis shows that the NUDT9H domain for both channels is significantly different to the human NUDT9 enzyme as well as to the NUDT9H domain of nvTRPM2 [e.g., 68]. This applies in particular to the sequence of the Nudix box, which is crucial for the catalytic activity of NUDT9 [15]. In line with this, it has been demonstrated that the NUDT9H domain of hTRPM2 does not exhibit significant ADPRase activity although it can effectively bind ADPR [2,18,68]. Moreover, the data from the latest cryo-EM analysis of hTRPM2 underline the important role of the NUDT9H domain as an additional binding pocket for ADPR [25]. For the NUDT9H domain of drTRPM2 on the other hand, it was shown that even the binding affinity to ADPR is dramatically reduced [68] and thus ADPR-dependent channel activation is most probably performed via the N-terminal binding pocket [26]. When ADPR-binding to NUDT9H does no longer play a significant role, what kind of essential function does the NUDT9H domain have in drTRPM2?

The present cryo-EM structures of drTRPM2 and hTRPM2 suggest a different interaction between NUDT9H and channel domain [24,25,68]. In this context, apparently a specific sequence of NUDT9H which is missing in drTRPM2 plays a crucial role [68]. In hTRPM2 this so-called P-loop located within the N-terminal part of NUDT9H [68] was identified as a putative binding site for calmodulin and was associated with temperature-dependent channel activation [74].

A possible approach to investigate the species-specific importance of NUDT9H is the functional characterization of TRPM2 chimeras where this domain has been exchanged. Such an analysis was first carried out with hTRPM2 and nvTRPM2 [57]. The obtained results provided a first clue for a NUDT9H-independent channel activity of nvTRPM2 as well as of specific interdomain interactions in hTRPM2, which cannot be compensated for by an exogenous NUDT9H domain [16,57]. Based on the data derived from the cryo-EM structures, one should expect that corresponding chimeras of drTRPM2 and hTRPM2 are non-functional, since the structure-function relationship between NUDT9H and channel domain is distinctly different in both species. Surprisingly, the functional analysis revealed that ADPR-directed activation of drTRPM2-hNUD is quite similar to the corresponding wild-type channel (drTRPM2) [26]. In contrast, the reverse chimera hTRPM2-drNUD only sporadically responded to ADPR, irrespective of the agonist concentration used [26]. In this regard, further experiments have to be carried out in order to clarify this issue.

Remarkably, it was shown that the exchange of a single amino acid residue (Asn to Asp) in a highly conserved sequence motif of the NUDT9H domain induces a loss of function phenotype both in drTRPM2 [26] and hTRPM2 [19]. The corresponding point mutation in nvTRPM2 most probably suppressed the ADPRase activity of NUDT9H, since the channel mutant shows the characteristic gain-of-function phenotype with regard to the sensitivity to hydrogen peroxide [16]. Since the affected asparagine residue most likely is not directly involved in ADPR-binding, it could play a critical role in the functional structure of both ADPRase and interdomain interactions.

From an evolutionary perspective, the functional role of the NUDT9H domain seems to have changed significantly during the development of multicellular organisms. Originally representing a cytosolic or mitochondrial housekeeping ADPRase, the ancient NUDT9 homolog had most probably regulatory functions after fusion with a TRPM2 channel precursor. Thus, channel activity of the resulting chanzyme could have been controlled via the intracellular ADPR concentration, while there was no direct participation of NUDT9H in the gating mechanism of TRPM2. In hTRPM2, the function of NUDT9H lastly is reduced to a mere ADPR binding pocket without ADPRase activity, but at the same time it gained critical control over channel gating. According to a hypothesis of Iordanov et al. [71] in the course of evolution the loss of catalytic function of NUDT9H was accompanied by a drastic change in the stability of the channel pore. The experimental finding that suppression of ADPRase activity in nvTRPM2 induces sensitivity to oxidative stress [16,57] may directly reflect this evolutionary process. Accordingly, the present physiological role of hTRPM2, as a bona fide sensor for oxidative stress and crucial mediator of cellular apoptosis, could only develop after the loss of its endogenous ADPRase function.

## 7. Open Questions and Future Strategies

Despite the striking progress in determining the protein structure of TRPM2 as well as fast growing data on the functional properties of the novel ADPR binding pocket, the gating mechanism of TRPM2 has not yet been satisfactorily elucidated. This is primarily due to the pronounced species-specific differences that have been discovered so far. There is, for example, the NUDT9H domain, in which both the protein sequence and the functional role were significantly changed in the course of evolution [59,71]. However, this does not apply to the N-terminal ADPR binding pocket. Here, the critical amino acid residues are highly conserved in distantly related species variants such as in humans and sea anemones, even if the corresponding substrate specificities are significantly different [46,70]. In fact, this finding raises some concerns, especially since mutations in the supposed ADPR binding pocket do not only have effects on ADPR binding but also drastically change sensitivity to 2-APB [26]. Thus, additional cryo-EM studies should investigate the species-specific interactions between 2-APB and TRPM2 in more detail. It is quite possible that there is a close relationship between the gating mechanisms induced by ADPR and 2-APB as previously proposed [61].

Altogether, the currently available data strongly suggest that the N-terminal binding pocket may not be sufficient for the ADPR-dependent activation of TRPM2. Even in the case of nvTRPM2, which is activated independently of NUDT9H [16], further channel domains could be of critical importance for ADPR-dependent channel gating. This is all the more true for the TRPM2 orthologues from zebrafish and human, since here the presence of the NUDT9H domain is indispensable [16,24]. Perhaps, the characterization of the functional cooperation between NUDT9H and channel domain of TRPM2 in vertebrates may provide valuable information to identify the additional but yet unknown activation domain(s). Further cryo-EM studies have to be carried out in order to identify all the amino acid residues involved. For this purpose protein chimeras should also be used, in which different NUDT9H domains are combined with one and the same channel domain. This approach would be particularly interesting for nvTRPM2, since its functionally and structurally independent NUDT9H domain could not be defined in the cryo-EM structure [39]. Possibly, this goal may be better achieved with a NUDT9H domain adopted from vertebrate TRPM2, since it is more suitable for interdomain interactions. The experimental finding that the N-terminal binding pocket can differentiate between ADPR and IDPR [70] could be used as a tool to investigate the critical amino acids for their actual functional significance. The aim of such an investigation would be to reverse the substrate specificity of the N-terminal binding pocket for ADPR and IDPR by targeted mutagenesis.

Currently, one of the most pressing questions is the experimental verification of the hypothesis that two ADPR molecules must bind to activate hTRPM2. As described in Section 5, there are some experimental findings that are difficult to reconcile with this hypothesis. In particular, the experimental data obtained so far strongly suggest that the proposed mechanism does not apply to nvTRPM2 and drTRPM2. Due to the inherent snapshot characteristics of the cryo-EM method, it is absolutely necessary to carry out further functional studies in order to test this hypothesis.

Due to the fact that Ca^2+^ represents a cofactor for channel activation of TRPM2, a potential regulatory role for calmodulin has also been suggested. For hTRPM2 there is some experimental evidence for this idea, both at the sequence level and in functional respect [36,74]. Further studies in particular cryo-EM-analyses are necessary to clarify this issue.

For most of the TRPM subfamily investigated so far, PIP_2_ plays an important role in controlling channel activity [40,41]. In light of the species-specific variations of the gating mechanism of TRPM2, further investigations are necessary to pinpoint the structure-function relationship between the channel and PIP_2_. The currently available cryo-EM structures of TRPM2 were created in the presence of detergents, which means that in principle no reliable statements could be made about protein–lipid interactions. Therefore, future structural studies on the interaction between TRPM2 and PIP_2_ should be performed with the protein embedded in a lipid nanodisc. It is believed that further refined experimental approaches will lead to a model of the ADPR-dependent activation of TRPM2, which can also explain species-specific differences in an appropriate manner.

Because of its property to serve as an adjustable gateway for Ca^2+^ into the cell, TRPM2 is well-established in numerous physiological functions e.g., [9,10,11,12,13]. However, which additional physiological effects TRPM2 induces, e.g., through interaction with other cellular factors, is still unclear. In this context, species-specific differences could also contribute significantly to clarify this issue. In particular, studies on the well-established model organism *Nematostella vectensis* could provide important information on the elementary physiological importance of TRPM2 and NUDT9H. One could, for example, perform knock-out experiments in which one of the two functionally independent domains is selectively switched off. It would be also of great interest to specify the expression pattern of this channel in *Nematostella vectensis* at different stages of development.

All in all, there are still numerous questions regarding the gating mechanism and the physiological role of TRPM2, which are waiting to be answered with appropriate experimental approaches.

## Figures and Tables

**Table 1:** Overview of the currently available cryo-EM (cryogenic electron microscopy) data of different species variants of the TRPM2 channel. This includes the respective structure resolutions, the verification of the N-terminal or C-terminal ADPR (ADP-ribose) binding sites, as well as the main structural and functional results

Cryo-EM Study	Resolution of Cryo-EM	Nterm-ADPR (MHR 1/2)	Cterm-ADPR (NUDT9H)	Structural Features	Functional Features
***Nematostella vectensis*** [39] **EMD:** 7542 **PDB:** 6CO7	~3 Å	Not identified	Not identified	Pore, Ca^2+^-binding sites	Regulation by Ca^2+^ + PIP_2_
***Danio rerio*** (**I**) [24] **EMD:** 7999/8901 **PDB:** 6DRK/6DRJ	3.3-3.8 Å	Identified	Not identified	Apo-structure (EDTA) Active state (ADPR/ Ca^2+^) MHR1/2: ligand sensing MHR 3/4: signal transduction	MHR 1/2: Alanine scanning mutagenesis. Patch-clamp and surface expression
***Homo sapiens*** (**I**) [68] **EMD:** 9132-9134 **PDB:** 6MIX/6MIZ/6MJ2	3.6-6.1/6.4 Å	Not identified	Identified	Rigid body rotation of NUDT9H and MHR 1/2 affect ADPR binding	drTRPM2 vs. hTRPM2: ADPR-affinity of NUDT9H P-loop difference MHR cis interactions MHR trans interactions
***Homo sapiens*** (**II**) [25] **EMD:** 20478-20480/20482 **PDB:** 6PUO/6PUR/6PUS/6PUU	3.3-4.4 Å	Identified	Identified	ADPR must bind to both binding pockets for channel activation. 8-Br-cADPR selectively binds to MHR 1/2 and locks channel in Apo state	MHR 1/2: Alanine scanning mutagenesis. Patch-clamp and surface expression
***Danio rerio*** (**II**) [69] **EMD-**20367/20368/7822/20369 **PDB:** 6PKV/6PKW/6D73/6PKX	3.8-4.2 Å	Identified	Not identified	Twofold symmetric intermediate states quaternary structural rearrangements	No functional studies

MHR: Mitochondrial homologous recombination protein.

**Table 2:** Substrate specificities of the two ADPR binding pockets in different species variants of TRPM2 (transient receptor potential melastatin type 2 cation channel) as derived from the currently available data. Annotations that are derived indirectly without being experimentally confirmed have a question mark. The N-terminal ADPR binding pocket is synonymous with MHR 1/2 and the C-terminal ADPR binding pocket is synonymous with NUDT9H; n.d. not determined; Ago. agonist; Ant. Antagonist.

Compound	NvM2-MHR1/2	NvM2-NUDT9H	DrM2-MHR1/2	DrM2-NUDT9H	hM2-MHR1/2	hM2-NUDT9H
**ADPR**	Activation [16]	Hydrolysis [27]	Cryo-EM (Ago.) [24]	Weak binding [68]	Cryo-EM (Ago.) [68]	Cryo-EM (Ago.) [68]
**ADPR-2P**	Activation [16]	Hydrolysis [27]	n.d.	n.d.	Ligand to activate?	Ligand to activate?
**2d-ADPR**	Activation [27]	Hydrolysis [27]	n.d.	n.d.	Ligand to activate?	Ligand to activate?
**8-Br-ADPR**	Activation [27]	Hydrolysis [27]	n.d.	n.d.	Ligand to inhibit?	Ligand to inhibit?
**8-TP-ADPR**	Activation [70]	n.d.	Activation [26]	Ineffective binding?	Ligand to inhibit?	Ligand to inhibit?
**8-(3AcPhe)-ADPR**	Activation [70]	n.d.	Activation [26]	Ineffective binding?	Ligand to inhibit?	Ligand to inhibit?
**IDPR**	Activation [70] (via ADPR)	Competitive inhibition of ADPRase activity [70]	No effect [26]	Ineffective binding?	No interaction?	Activation [70]
**ε-ADPR**	Inhibition [27]	Hydrolysis [27]	n.d.	n.d.	n.d.	n.d.
**AMPCPR**	Activation [71]	No hydrolysis [71]	n.d.	n.d.	Ligand to activate?	Ligand to activate?
**8-Br-cADPR**	n.d.	n.d.	n.d.	n.d.	Cryo-EM (Antag.) [25]	No interaction [25]
**cADPR**	n.d.	n.d.	n.d.	n.d.	n.d.	Activation [34]

## Abbreviations

TRPM2	Transient receptor potential melastatin type 2
ADPR	Adensoine diphospho ribose
AMPCPR	α-β-methylene-ADPR
NUDT9H	Nucleotide diphosphate linked moiety X type motif 9 homology domain
PIP_2_	Phosphatidylinositol-4,5-bisphosphate
ADPRase	ADPR hydrolase 9
2-APB	2-Aminoethoxydiphenyl borate
IDPR	Inosine diphospho ribose
NAD^+^	Nicotine amide adenine dinucleotide
EMDB	Electron microscopy data bank
PDB	Protein data bank
